# Beyond the Malnutrition Screening Tool: Assessing Hand Grip Strength and Gastrointestinal Symptoms for Malnutrition Prediction in Outpatients with Chronic Kidney Disease Not on Kidney Replacement Therapy †

**DOI:** 10.3390/nu17152471

**Published:** 2025-07-29

**Authors:** Maya Young, Jessica Dawson, Ivor J. Katz, Kylie Turner, Maria Chan

**Affiliations:** 1 Department of Nutrition and Dietetics, St George Hospital, Kogarah, NSW 2217, Australia; maya.young@health.nsw.gov.au (M.Y.); maria.chan@health.nsw.gov.au (M.C.); 2 NHMRC Clinical Trials Centre, University of Sydney, Sydney, NSW 2006, Australia; 3 Department of Renal Medicine, St George Hospital, Kogarah, NSW 2217, Australia; ivor.katz@health.nsw.gov.au (I.J.K.); kylie.turner1@health.nsw.gov.au (K.T.)

**Keywords:** malnutrition, chronic kidney disease, malnutrition screening tool, gastrointestinal symptoms, hand grip strength

## Abstract

**Background**: The Malnutrition Screening Tool (MST) is commonly used to identify malnutrition risk; however it has demonstrated poor sensitivity to detect malnutrition in inpatients with chronic kidney disease (CKD) and kidney replacement therapy (KRT) populations. Gastrointestinal symptoms, such as poor appetite, may better detect malnutrition. The accuracy of MST or other nutrition-related parameters to detect malnutrition in ambulatory patients with CKD stages 4–5 without KRT has not been evaluated. **Methods**: A single site retrospective audit of outpatient records from May 2020 to March 2025 was conducted. Patients with eGFR < 25 mL/min/1.73 m^2^ without KRT who had both MST and a 7-point Subjective Global Assessment (SGA) within 7 days were included. Sensitivity, specificity, and ROC-AUC analyses compared nutritional parameters against SGA-defined malnutrition. Nutritional parameters tested included MST, hand grip strength, upper gastrointestinal symptom burden, poor appetite and a combination of some of these parameters. **Results**: Among 231 patients (68.8% male, median age 69 years, median eGFR 15), 29.9% were at risk of malnutrition (MST ≥ 2) and 33.8% malnourished (SGA ≤ 5). All potential screening tools had AUC ranging from 0.604 to 0.710, implying a poor-to-moderate discriminator ability to detect malnutrition. Combining HGS ≤ 29.5 kg or MST ≥2 demonstrated high sensitivity (95.5%) and negative predictive value (93.3%), but low specificity (33.3%) for detecting malnutrition, indicating this approach is effective for ruling out malnutrition but may over-identify at-risk individuals. **Conclusions**: MST and other tested tools showed limited overall accuracy to identify malnutrition. Using combined nutritional markers of HGS or MST score was the most sensitive tool for detecting malnutrition in this advanced CKD without KRT population.

## 1. Introduction

Malnutrition is common in chronic kidney disease (CKD), with global prevalence estimates of 42.7% (35.2–60%) [1]. It increases in prevalence as kidney disease progresses [2]. The identification and treatment of malnutrition in CKD is vital, as malnutrition is associated with adverse patient outcomes, reduced quality of life, increased rate of hospitalization and is an independent predictor of mortality [3,4,5]. Nutrition guidelines for CKD recommend bi-annual screening for malnutrition using a validated nutrition screening tool, but do not identify which screening tool to use [6].

An appropriate nutrition screening tool needs to be able to accurately identify people who are at risk of malnutrition [7], as this often prompts referral to a dietitian. A dietitian is trained in implementing medical nutrition therapy to prevent the development or progression of malnutrition. Many of the conventional nutrition screening tools include unintentional weight loss as a primary indicator of malnutrition [7]. However, in CKD, and particularly once in kidney failure, assessment of weight changes is difficult due to fluid shifts and fluid overload. Conventional nutrition screening tools are also impacted by physiological changes, as often reduced intake may be associated with spontaneous reduction in physical activity to conserve body mass, hence little to no weight loss may be observed, relying on the parallel use of other indicators to determine malnutrition [8]. The Malnutrition Screening Tool (MST) is a commonly used tool to screen for malnutrition that is validated in inpatient and outpatient settings for various clinical conditions [9]. However, data shows the MST is not sensitive enough to identify malnutrition in renal inpatients [10]. Only one study evaluated the accuracy of malnutrition screening (using the Malnutrition Universal Screening Tool) in both inpatients and outpatients with CKD not receiving kidney replacement therapy [11]. However, this study did not report the tool’s accuracy separately for inpatients versus outpatients. Nutrition-related symptoms (such as poor appetite, taste changes, and nausea) are highly prevalent in kidney disease, with some data indicating that the presence of nutrition related symptoms may be better at detecting malnutrition in CKD [11,12]. A study conducted in peritoneal dialysis patients demonstrated that appetite loss was a key predictor for malnutrition [13].

Whether the MST accurately identifies malnutrition in outpatients with CKD without kidney replacement therapy (KRT) has not been evaluated. Therefore, the primary aim of this study is to examine if the MST is a sensitive, specific, and accurate malnutrition screening tool in outpatients with CKD without KRT. A secondary aim of the study is to determine the accuracy of other nutritional markers, including loss of appetite, gastrointestinal (GI) symptom burden, and handgrip strength to predict malnutrition in outpatients with CKD without KRT.

## 2. Materials and Methods

This study was a cross-sectional retrospective audit of electronic medical records of people attending a kidney disease education clinic (KDEC) at an Australian tertiary teaching hospital. This multidisciplinary clinic includes people with advanced kidney disease who are making choices about their treatment pathway and are not currently receiving KRT. This nurse-led clinic includes a dietitian and social worker. Patients are routinely seen by both the nurse and dietitian, where the nurse screens patients using the MST and iPOS-Renal symptom survey, and the dietitian independently performs the validated 7-point Subjective Global Assessment (SGA) [14]. Data collection took place over a 5-year period from 1 May 2020 to 31 March 2025. All patients who, on initial clinic appointment, had an MST and a 7-point SGA completed within 7 days of each other, were included. The participants were considered well-nourished if they scored ≥ 6, and they were considered malnourished if they scored ≤ 5. The data was extracted from electronic medical records and the clinic patient database; it is maintained as part of routine clinical practice. The data collected included age, gender, ethnicity, biochemistry (eGFR, creatinine, albumin), primary renal disease, MST score and date, weight, height, Body Mass Index (BMI), iPOS-Renal symptom scores, and hand grip strength (HGS). The HGS measurements were conducted using the Jamar Digital Dynamometer (S.I. Instruments) on the dominant arm, of the non-fistula arm, if applicable, with the patient in a seated position with the arm bent at 90 degrees. Two HGS measures were conducted with the average of these two readings recorded. For iPOS-Renal scores, only the GI symptom scores were included for analysis, as previous research associates these symptoms with malnutrition in kidney disease cohorts [15,16]. These GI symptoms included diarrhea, constipation, nausea, vomiting, poor appetite, and mouth problems. Upper GI symptoms were further refined to include nausea, vomiting, poor appetite, and mouth problems. The iPOS-Renal asks patients to rate symptoms on a scale from 0 (none) to 4 (overwhelming) over the past 7 days. Overall GI symptom severity was calculated by adding the total scores for each symptom (0–4) and dividing by the total number of GI symptoms reported. As there is no single universally standardized method for calculating HGS that integrates gender, age, and other factors into a single formula, for this study, we used crude values that enabled a cut-off point to be determined in ROC-AUC analyses. Ethics were approved by the South-Eastern Sydney Local Health District Human Research Ethics Committee (2025/ETH00667).

The data was analyzed using SPSS [Version 29.0.1.0]. The demographic data was determined using descriptive statistics. Chi squared tests were used to determine the difference between well-nourished and malnourished cohorts for categorial variables, while Student’s t-test was used to analyze normally distributed continuous variables, or Mann–Whitney for non-parametric continuous variables. SGA and MST were collapsed into two categories for analysis. SGA score ≥ 6 was considered well-nourished and ≤ 5 malnourished. The MST ≥ 2 was considered “at risk of malnutrition”, and MST < 2 was considered “not at risk of malnutrition”. This classification method of the SGA has been shown to be both valid and reliable [17]. Binary logistic regression analysis to investigate predictors of malnutrition was conducted with variables that showed significant (*p* < 0.05) associations with malnutrition. Assumptions of multicollinearity were assessed, with variables with a variance inflation factor (VIF) <2.5 included in the model. Contingency tables were used to compare SGA and MST data, appetite scores, and other nutritional parameters, and to calculate sensitivity, specificity, positive predictive value (PPV), and negative predictive value (NPV). Receiver operator curve-area under the curve (ROC-AUC) score was used to calculate overall accuracy of MST, appetite, and other nutritional parameters at detecting malnutrition. Statistical significance was defined as *p* < 0.05.

## 3. Results

**Figure 1:**
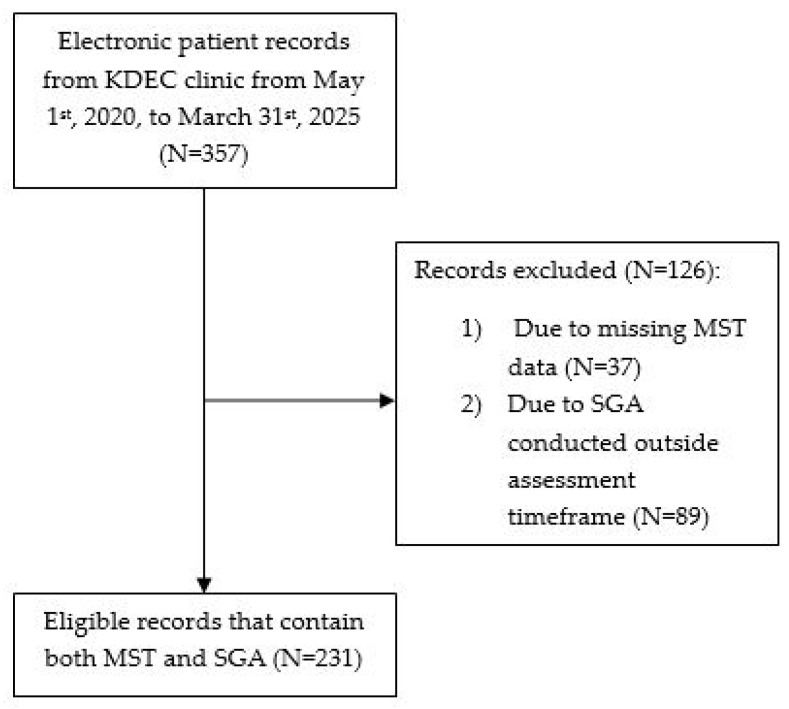
Flowchart of study population.

In total, 357 records were extracted. Of these, 37 records did not have an MST, and a further 89 participants did not have an SGA conducted within the assessment timeframe (Figure 1). The final analyses included 231 records that contained both MST and SGA. Most of the cohort were male (68.8%) with a median age of 69 years and median eGFR of 15 mL/min/1.73 m^2^ (Table 1). The primary causes of renal disease were diabetes mellitus (35.9%), hypertension (16.5%), and glomerular pathophysiology (16.5%). In total, 29.9% were assessed as being at risk of malnutrition (MST ≥ 2), while 33.7% of the cohort were assessed as being malnourished (7-point SGA ≤ 5) (Table 1).

Table 2 outlines the characteristics of malnourished and well-nourished patients. People who were malnourished were older, more often female, and had lower eGFR, body weight, BMI, and HGS than those assessed as being well-nourished. Compared to well-nourished patients, rates of GI symptoms were higher in the malnourished cohort, with a median of 2 GI symptoms (IQR 1–3) that were rated as more severe with a median score of 1.5 (1–2) indicating symptoms were rated as slight-to-moderate. The GI symptoms that were more common in malnourished patients were mouth problems (54.5% vs. 39.4%; *p* = 0.042), poor appetite (50.7% vs. 25.4%; *p* = <0.001), and vomiting (11% vs. 2.8%; *p* = 0.013). The MST and HGS predicted malnutrition, independent of upper GI symptom burden, age, and gender (Table 3).

Table 4 reports the ROC-AUC of MST and other nutritional parameters for detecting malnutrition. The MST ≥ 2 and poor appetite ≥1 demonstrated modest accuracy for detecting malnutrition, with ROC-AUC values of 0.63 and 0.63, respectively. The MST had a false positive (FP) count of 32 and a false negative (FN) count of 41, resulting in a false positive rate (FPR) of 20.9% and false negative rate (FNR) of 52.6%. Poor appetite had 36 FPs and 35 FNs with FPR of 24.7% and FNR of 48.6%. Compared to poor appetite alone, using upper GI symptom presence modestly improved sensitivity to detect malnutrition (51.4% vs. 77.8%, respectively); however, specificity declined (74.8% vs. 46.9%, respectively). Using upper GI symptoms resulted in 76 FPs and 16 FNs, with FPR of 53.2% and FNR of 22.2%. When evaluating HGS, a threshold of 29.5 kg indicated the best balance of sensitivity (84.1%) and specificity (45.2%) and showed the highest individual discriminatory ability (ROC-AUC 0.71) to detect malnutrition. Using HGS ≤ 29.5 kg, the FP and FN counts were 46 and 7, respectively, resulting in a FPR of 54.8% and FNR of 15.9%. Combining HGS ≤ 29.5 kg and MST ≥ 2 did not improve overall accuracy (ROC-AUC 0.60), while using HGS ≤ 29.5 kg or MST ≥ 2 increased sensitivity substantially (96%) but reduced specificity (33%), indicating a higher rate of false positives. The combination approach also yielded the highest negative predictive value (93%), suggesting strong utility for ruling out malnutrition. When using both HGS and MST, there were 13 FPs and 28 FNs (FPR of 15.5% and FNR of 63.6%), whilst using HGS or MST resulted in 56 FPs and 2 FNs (FPR of 66.7% and FNR of 4.5%).

## 4. Discussion

The MST (score ≥ 2) identified 29.9% of this renal outpatient population being at risk of malnutrition, whilst malnutrition was diagnosed (7-point SGA score ≤ 5) in 33.7% of the population. Malnutrition was associated with older age, female sex, lower body weight, BMI, and reduced HGS. The MST and HGS were predictors of malnutrition independent of eGFR, age, sex, and upper GI symptom burden. However, the use of MST, upper GI symptom burden, and HGS as nutrition screening tools all demonstrated a poor-to-moderate ability to detect malnutrition, with questionable value in clinical practice. The combination of HGS (≤29.5 kg) or MST (score ≥ 2) demonstrated very high sensitivity (95.5%) but high rates of false positives. The high negative predictive value score (71.1%) means that this tool would effectively rule out those who are well nourished. In under-resourced clinical settings, nutrition screening provides a quick way to identify patients who need dietetic intervention. However, screening tools with high false-positive rates will generate unnecessary referrals and waste limited resources. Identification of an appropriate nutrition screening tool for people with advanced CKD without KRT is still needed.

This study reflects that the MST is not an accurate tool to detect malnutrition in an advanced CKD without KRT population. This study found the MST had a sensitivity of 47.4% and an overall accuracy of 63.3%. This is consistent with data from inpatient CKD populations that report the MST having a sensitivity of 48.7% (95% CI 0.417, 0.540) and overall accuracy of 66.2% (95% CI 0.589, 0.718) [10]. A systematic review of the validity of nutrition screening tools in all hospitalized patients, reported that when referencing against the SGA, the MST had sensitivity of 0.81% (95% CI 0.67, 0.90) and specificity of 0.79% (95% CI 0.72, 0.84) [18]. Similarly, the Malnutrition Universal Screening Tool (MUST) showed sensitivity of 0.84 (95% CI 0.73, 0.91) and specificity of 0.85 (95% CI 0.75, 0.91) when referenced against the SGA. Whilst these results indicate better sensitivity and specificity in heterogeneous populations, it was noted both the MST and MUST performed poorly in kidney disease populations [18]. The MST and other commonly used nutrition screening tools rely heavily on changes in body weight to determine nutritional risk. ESPEN guidelines on Clinical Nutrition in Hospitalized People with Acute or Chronic Kidney Disease highlight body weight and body mass index to be poor markers of nutritional status due to fluid accumulation often masking changes in dry or body weight [8].

Other screening tools have been proposed to identify malnutrition in CKD populations. The Renal inpatient nutrition screening tool (Renal iNUT) incorporates additional questions related to appetite, oral intake and existing use of oral nutrition supplements, and has a sensitivity ranging between 59.4 and 92.8% and specificity between 54 and 92.3%, depending on the cut-off value used [19,20,21,22]. Therefore, this tool shows some improvements compared to MST in renal inpatients and MUST in people receiving haemodialysis Turkey. However, this tool has not been validated in outpatients with CKD. In addition, the Geriatric Nutrition Risk Index (GRNI) tool has been tested in peritoneal dialysis (PD) patients; it shows a sensitivity of 54.4% and specificity of 71.1%, suggesting that it is a poor tool to identify nutrition risk in PD ambulatory patients [23]. Other tools, such as the Simple Nutrition Screening Tool (SNST) and Nutrition Risk Screening (NRS), showed AUC 0.847 (vs. 0.749, respectively) when referenced against the SGA in a maintenance haemodialysis population in Indonesia [24]. The SNST assesses six visual clues of a person’s nutritional status but has only been validated in a single Indonesian inpatient population [25]. The NRS assesses changes in weight and stress factors. Further validation of whether these tools can accurately predict malnutrition in other renal populations is needed.

The use of GI symptoms to screen for malnutrition has gained momentum in populations with kidney disease. In renal inpatients, the use of the nutrition impact score (NIS) component of the Patient-Generated SGA (PG-SGA) demonstrated good accuracy to detect malnutrition (AUC 0.81, 95% CI 0.74, 0.88) [12]. Similar results were produced using the NIS to detect malnutrition in haemodialysis patients (AUC 0.86, CI 0.79–0.93) [26]. In the current study, GI symptom burden showed only poor or moderate ability to detect malnutrition. Of note, the NIS component of the PG-SGA assesses symptoms that affect a person’s ability to eat adequately, whilst the iPOS-renal does not report the impact on a person’s eating or adequacy of intake. In a retrospective analysis of 227 people with moderate to advanced CKD (mean eGFR 17 mL/min/1.73 m^2^), self-reporting appetite on a 5-point Likert scale showed that rating good-very good appetite had a high PPV of 0.92 (95% CI 0.88–0.95) for predicting adequate protein intake but not energy intake (AUC 0.42, 95% CI 0.36–0.45) [27]. Evaluation of whether the NIS component of the PG-SGA may better detect malnutrition in outpatient settings is warranted.

Use of HGS as a simple and an indirect measure of nutritional status and functional status is recommended in the latest KDOQI CKD Nutrition Guidelines [6]. Compared to the other nutritional parameters assessed in the current study, HGS demonstrated highest accuracy to detect malnutrition (AUC 0.71), with a reasonable sensitivity (84.1%) but low specificity (45.2%). This is reflected in another study of CKD without KRT that demonstrated HGS AUC (0.64 for men and 0.71 for women) [28]. However, in an elderly surgical inpatient population, HGS showed very poor accuracy to detect malnutrition (AUC 0.41) [29]. As CKD is increasingly prevalent in elderly people, future assessment of the accuracy of detecting malnutrition in elderly CKD is warranted.

To our knowledge, this study presents novel findings regarding the accuracy of various commonly measured nutritional parameters to detect malnutrition in outpatients with advanced CKD without KRT. Another strength is the use of a CKD-validated nutrition assessment tool (7-point SGA) to diagnose malnutrition that was conducted by only one clinician, reducing inter-rater variability. Limitations of this study include an observational, cross-sectional design, as such a causal relationship between these nutritional parameters and nutritional status cannot be established. Second, the presence of fluid overload and other clinical conditions of the cohort was not recorded, limiting inferences of the impact of this on accuracy of screening tools. Third, HGS was not referenced against sex, handedness, BMI, or age cut-offs, as this was a retrospective analysis, and not all data was available. Finally, this study was conducted in a single site with a predominantly Caucasian population, so further studies in other populations are required to confirm our findings and expand the generalizability of our findings.

Future research should assess the accuracy of symptom assessment tools, such as NIS (from PG-SGA), that have shown good ability to detect malnutrition in renal inpatients [12] and hemodialysis populations [26] in CKD outpatient populations. Future studies should evaluate whether factors such as age, sex, BMI, co-morbidities, or handedness cut-offs can improve the sensitivity of HGS in malnutrition screening. In addition, whether biochemical parameters may further improve identification and management needs further evaluation; however, cost and patient burden need to be considered.

## 5. Conclusions

Overall, all screening tools and combinations evaluated in this study demonstrated only poor-to-moderate ability to discriminate malnutrition, with trade-offs between sensitivity and specificity depending on the approach used. Our findings suggest that while MST is a practical screening tool, incorporating objective measures such as HGS enhances malnutrition detection in patients with kidney disease. The implementation of MST and HGS for nutrition screening needs to consider available resourcing, including appropriate staffing and training as well as access to specialist equipment such as hand dynamometers. Combining subjective and objective assessments may improve early identification and timely nutritional interventions in this high-risk population. Future studies are needed to identify an accurate and simple tool to detect malnutrition in ambulatory outpatients with advanced CKD without KRT.

## Figures and Tables

**Table 1:** Participant characteristics (*n* = 231).

Sex, male (%)	159 (68.8)
Age, years (median, IQR)	69 (58–76)
Ethnicity, Caucasian (%)	148 (64.1)
Primary cause of kidney disease (%)	
Diabetes Mellitus	83 (35.9)
Glomerular Disease	38 (16.5)
Hypertension/Vascular Disease	38 (16.5)
Hereditary Disease	25 (10.8)
Tubulointerstitial Disease	14 (6.1)
Other	11 (4.8)
Not reported	22 (9.5)
Anthropometry (mean, SD)	
Weight, kg	81.7 (28.0)
Body Mass Index, kg/m^2^	28.8 (8.4)
Biochemistry (median, IQR)	
eGFR, ml/min/1.73 m^2^	15 (12–18)
Creatinine, umol/L	326 (280–391)
Albumin, g/L	37 (32–41)
MST, score ≥ 2 (%)	69 (29.9)
Nutritional status, malnourished SGA score ≤ 5 (%)	78 (33.7)

eGFR—estimated glomerular filtration rate; MST—malnutrition screening tool.

**Table 2:** Patient characteristics classified by nutritional status.

Participant Characteristics	Well Nourished (*n* = 153)	Malnourished (*n* = 78)	*p* Value
Sex, male (%)	113 (73.8)	46 (58.9)	0.025
Age, median (IQR)	67 (56–75)	74 (62–78)	0.002
Anthropometry			
Weight, kg, mean (SD)	90.2 (23.5)	70.7 (16.9)	<0.001
BMI, kg/m^2^, mean (SD)	31.2 (7.6)	25.9 (5.5)	<0.001
Handgrip Strength, kg, median (IQR) *	28.0 (23.5–36.5)	23.0 (18.0–27.0)	<0.001
Biochemistry (IQR)			
eGFR, mL/min/1.73 m^2^	15 (13–18)	14 (11–17)	0.033
Creatinine, umol/L	326 (285–390)	328 (279–394)	0.967
Albumin, g/L	37 (33–41)	37 (32–41)	0.887
Symptoms **			
Poor appetite (%)	35 (25.2)	37 (51.3)	<0.001
Poor appetite severity, median (IQR)	1 (1–2)	2 (1–2)	0.463
Mouth Problems (%)	57 (39.8)	39 (54.2)	0.059
Mouth Problems severity, median (IQR)	1 (1–2)	2 (1–2)	0.412
Nausea (%)	28 (19.5)	20 (27.8)	0.224
Nausea severity, median (IQR)	1 (1–2)	1 (1–2)	0.346
Vomiting (%)	4 (2.8)	8 (11.1)	0.023
Vomiting severity, median (IQR)	2 (2–2.5)	1 (1–1)	0.005
Constipation (%)	39 (27.2)	22 (30.5)	0.633
Constipation severity, median (IQR)	2 (1–2)	1 (1–2)	0.736
Diarrhea (%)	12 (8.4)	11 (15.3)	0.160
diarrhea severity, median (IQR)	1 (1–2)	1 (1–2)	0.786
Number of GI symptoms, median (IQR)	1.0 (0–2)	2.0 (1–3)	<0.001
GI symptom Severity, median (IQR)	1.0 (0–1.5)	1.4 (1–2)	<0.001
Number of upper GI symptoms, median (IQR)	1.0 (0–1.5)	1.0 (1–2)	<0.001
Symptom severity of upper GI symptoms, median (IQR)	1.0 (0–1.3)	1.0 (1–2)	<0.001

BMI—body mass index; eGFR—estimated glomerular filtration rate; GI—gastrointestinal; * Handgrip strength *n* = 128 (well-nourished *n* = 84, malnourished *n* = 44); ** Symptoms *n* = 215 (well-nourished *n* = 143, malnourished *n* = 72).

**Table 3:** Predictors of malnutrition.

Variable	Model 1		Model 2		Model 3		Model 4		Model 5	
	OR (95% CI)	*p* Value	OR (95% CI)	*p* Value	OR (95% CI)	*p* Value	OR (95% CI)	*p* Value	OR (95% CI)	*p* Value
MST	3.1 (1.9, 6.2)	<0.001	2.95 (1.3, 6.8)	0.011	2.9 (1.2, 6.8)	0.017	2.8 (1.2, 6.7)	0.018	2.7 (1.14, 6.6)	0.024
HGS			0.904 (0.7, 0.95)	<0.001	0.9 (0.8, 0.96)	<0.001	0.9 (0.9, 0.96)	0.001	0.9 (0.9, 0.98)	0.007
Upper GI presence					1.6 (1.1, 2.3)	0.01	1.5 (0.9, 2.5)	0.168	1.5 (0.9, 2.7)	0.153
Poor appetite							1.4 (0.4, 4.7)	0.62	1.1 (0.30, 4.2)	0.860
Age									1.0 (0.98, 1.1)	0.468
Gender									1.3 (0.5, 3.5)	0.615
eGFR									0.98 (0.9, 1.1)	0.846

Model 1: unadjusted (MST); Model 2: model 1 + HGS; Model 3: model 2 + upper GI; Model 4: model 3 + poor appetite; Model 5: model 4 + age, gender, eGFR; MST—malnutrition screening tool; HGS—handgrip strength; GI—gastrointestinal; eGFR—estimated glomerular filtration rate.

**Table 4:** Receiver operating characteristic (ROC) curve analysis of screening tools.

	MST ≥ 2 (*n* = 231)	HGS ≤ 29.5 kg (*n* = 128)	Poor appetite (*n* = 215)	Upper GI Symptom burden (*n* = 215)	HGS ≤ 29.5 kg and MST ≥ 2 (*n* = 128)	HGS ≤ 29.5 kg or MST ≥ 2 (*n* = 128)
**ROC-AUC (95%CI)**	0.633 (0.554, 0.750)	0.710 (0.615, 0.805)	0.631 (0.550, 0.712)	0.641 (0.564,0.719)	0.604 (0.498, 0.711)	0.644 (0.549, 0.739)
**Sensitivity (95%CI)**	47.4% (0.366, 0.585)	84.1% (0.715, 0.928)	51.4% (0.399, 0.627)	77.8% (0.673, 0.863)	50.9% (0.381, 0.636)	95.5% (0.866, 0.992)
**Specificity (95%CI)**	79.1% (0.722, 0.850)	45.2% (0.349, 0.559)	74.8% (0.679, 0.815)	46.9% (0.388, 0.550)	84.5% (0.758, 0.912)	33.3% (0.239, 0.438)
**PPV (95% CI)**	53.6% (0.419,0.651)	44.6% (0.342, 0.553)	50.7% (0.393, 0.620)	42.4% (0.342, 0.509)	69.0% (0.542, 0.816)	42.9% (0.333, 0.527)
**NPV (95% CI)**	74.7% (0.676, 0.810)	84.4% (0.721, 0.930)	75.7% (0.673, 0.822)	80.7% (0.714, 0.882)	71.7% (0.624, 0.800)	93.3% (0.808, 0.989)

ROC-AUC, receiver operating characteristic-area under the curve; PPV—positive predictive value; NPV—negative predictive value; MST—malnutrition screening tool; HGS—handgrip strength.

## Data Availability

The raw data supporting the conclusions of this article will be made available by the authors on request due to ethical reasons.

## Abbreviations

The following abbreviations are used in this manuscript:

AUC	Area Under the Curve
BMI	Body Mass Index
CI	Confidence Interval
CKD	Chronic Kidney Disease
eGFR	Estimated Glomerular Filtration Rate
ESPEN	European Society for Clinical Nutrition and Metabolism
GI	Gastrointestinal
GRNI	Geriatric Nutrition Risk Index
HGS	Hand Grip Strength
iNUT	Inpatient Nutrition screening tool
iPOS-Renal	International Palliative Care Outcome Scale Renal
KDEC	Kidney Disease Education Clinic
KDOQI	Kidney Disease Outcomes Quality Initiative
KRT	Kidney Replacement Therapy
MST	Malnutrition Screening Tool
MUST	Malnutrition Universal Screening Tool
NIS	Nutrition Impact Score
NPV	Negative Predictive Value
NRS	Nutrition Risk Screening
PD	Peritoneal Dialysis
PG-SGA	Patient-Generated Subjective Global Assessment
PPV	Positive Predictive Value
ROC	Receiver Operator Curve
SGA	Subjective Global Assessment
SNST	Simple Nutrition Screening Tool
SPSS	Statistical Package for the Social Sciences
VIF	Variance Inflation Factor
